# A case of postpsychotic depression improved by switching antipsychotic monotherapy

**DOI:** 10.1002/npr2.12308

**Published:** 2022-12-08

**Authors:** Yoshiyo Oguchi, Atsuo Nakagawa, Hiroki Kocha

**Affiliations:** ^1^ Department of Neuropsychiatry St. Marianna University School of Medicine Kawasaki Japan

**Keywords:** antipsychotics, depression, lurasidone hydrochloride, schizophrenia, suicide

## Abstract

**Aim:**

Postpsychotic depression is challenging to differentiate, yet it is clinically frequent, puts patients at risk for suicide, and affects their mental capacity. Treatment with antipsychotics for postpsychotic depression is desirable; however, there is no consensus on which antipsychotics are optimal.

**Case presentation:**

We report the case of a young male patient with schizophrenia in his 20s who developed postpsychotic depression, including despair, overwhelming loss, humiliation, and suicidal ideation during treatment with paliperidone. As a result, we switched his medication to lurasidone, which relieved his depressive symptoms without any symptom relapse and his social functioning improved.

**Conclusion:**

Postpsychotic depression has more psychic characteristics than behavioral. According to various international guidelines for the pharmacological treatment of schizophrenia, antipsychotics should be administered for depressive symptoms of schizophrenia. As evidenced in this case report, lurasidone may be a practical alternative for improving postpsychotic depression.

## Abstract 2

Lurasidone monotherapy improves postpsychotic depression in a young man.

## Graphical abstract

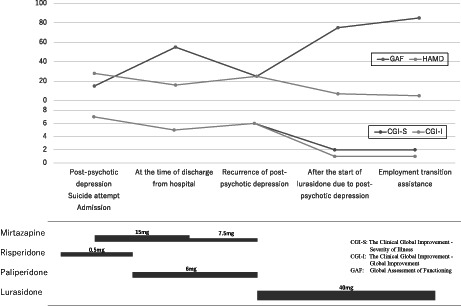

## 1 INTRODUCTION

Depressive symptoms in schizophrenia occur at all stages of the illness,^1^ with a prevalence ranging from 6% to 75% and a prevalence mode of 25%.^2^ Comorbid depressive symptoms can lead to social difficulties and increased suicide risk.^3^, ^4^

Postpsychotic depression (PPD) is depression that presents after the acute phase of schizophrenia. Mino and Ushijima^5^ refer to PPD as a “postpsychotic collapse” and indicate that it is a state of inactivity with a loss of energy and vitality after the improvement of psychotic symptoms. In his latest review,^6^ Guerrero stated, “We have always focused on hallucinatory delusions in patients with schizophrenia, but in the last two decades we have begun to reaffirm the importance of the emotional aspect as part of the recovery process in psychotic patients.”

The World Federation of Societies of Biological Psychiatry (WFSBP) guidelines suggest that in treating PPD, typical antipsychotics may cause a depressed or unpleasant mood depending on the degree of dopamine D2 blockade.^7^ In contrast, certain atypical antipsychotics may improve depressive symptoms.^7^, ^8^ Data on antidepressant administration for treating depressive symptoms of schizophrenia are limited.^7^ In recent The American Psychiatric Association practice guideline, there is evidence supporting the use of antidepressants to treat depression in people with schizophrenia; however, many trials had small sample sizes or other issues that raise the possibility of bias in the results.^8^, ^9^, ^10^, ^11^ Although concomitant use of antipsychotics and antidepressants can be useful for depressive state in schizophrenia, caution is needed due to drug interactions.^8^, ^12^ Thus, no precise evidence‐based drug therapy has been established.

Here, we report a case of a young adult male patient with schizophrenia with PPD, and a history of suicide attempts, who switched from paliperidone to lurasidone 40 mg, resulting in improvement of depressive symptoms characteristic of PPD and eventually able to lead to employment transition assistance.

## 2 CASE

A young man in his 20s, as a teenager, became hallucinatory and delusional exacerbated by friendship problems at school and first visited our clinic 12 years ago. We diagnosed him with schizophrenia, prescribed risperidone 0.5 mg, and his positive symptoms improved. Afterward, there was no recurrence; the patient remained stable for an extended period and worked after graduating from a university. However, 2 years ago, he became hallucinatory and delusional again following a problem at work, and his symptoms decreased after increasing risperidone to 3.5 mg. He quit his job 1 year ago and began attending daycare.

Three months after his resignation, he expressed concern about future employment at a daycare job search workshop. He felt depressed and lacked motivation but did not reach the point of depression. Soon, his pessimistic remarks, including “I had much to lose in my life because of my illness,” and “I couldn't find a job. I don't care anymore,” became more prominent, and he began feeling hopeless. Gradually, he could no longer attend daycare and began to withdraw to his room. He also began to complain of being “tired and sluggish” without reason and admitted to episodes of “not being able to hear someone's story through” and “not being able to do simple math.” However, there was no diurnal variation in agitation or mood.

After nearly 1 month of being in this condition, insomnia and suicidal thoughts began to occur, and a suicide attempt resulted in hospitalization. We prescribed mirtazapine 15 mg over risperidone 0.5 mg for postpsychotic depressive symptoms. Although fatigue remained, insomnia improved, and the lofty thoughts of death disappeared. Considering that he was making fewer pessimistic comments and wishes, he was discharged from the hospital after 2 weeks.

After discharge from the hospital, we discussed options with the patient and decided to replace risperidone with paliperidone 6 mg and continued mirtazapine 15 mg to resume attendance at daycare and switch to long‐acting injectable antipsychotic treatment. Soon, however, the patient was no longer able to attend daycare. Without giving any particular reason, he said, “The disease is no longer curable. It is humiliating” and continued to cover himself with a futon in his room. We judged this to be a relapse of PPD, as he again stated, “I have no hope for the future,” and had difficulty engaging in simple conversational interactions. Since these medications did not affect his depressive symptoms, we had him taper off mirtazapine, and switched from paliperidone to lurasidone 40 mg monotherapy.

Soon after the start of lurasidone, he said with a smile, “My sluggishness has gone away, and I have more motivation. I can see the brightness in front of my eyes.” He also began attending daycare again, could perform mental work at daycare, and think positively about employment. After discussions with relevant parties, he was employed at a labor transition support facility. He continues taking lurasidone 40 mg and is seeking to return to general employment.

## 3 DISCUSSION

Postpsychotic depression requires careful differential diagnosis, is frequent, and is characterized by psychological rather than behavioral features compared to depression.^6^ Suicide is a risk factor of PPD.^6^ Regarding pharmacotherapy, since the base of PPD is schizophrenia, we prefer continued administration of antipsychotics, which may improve depressive symptoms.^7^ In contrast, a Cochrane review by Whitehead et al.^13^ concluded that concomitant use of antidepressants did not significantly reduce depressive symptoms.

**FIGURE 1:**
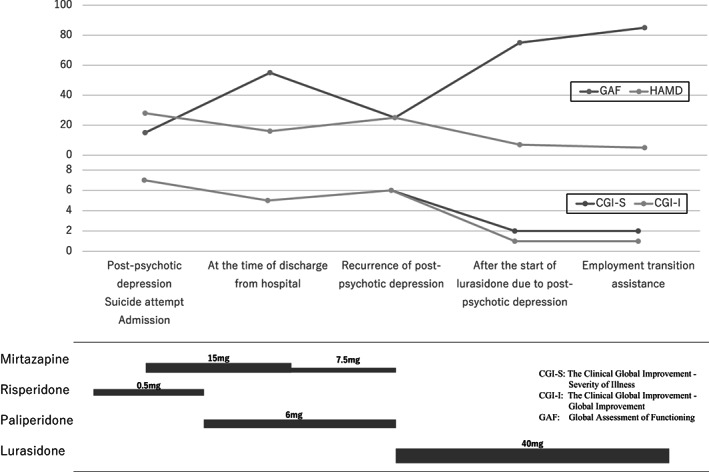
Clinical course and medications

We believe this case meets Birchwood's diagnostic criteria.^14^ The patient had moderate depression, with no concurrent psychotic symptoms. In addition, a subthreshold depressive episode preceded it. While typical antipsychotics are prone to produce PPD,^15^ this patient was taking atypical antipsychotics and unlikely to have experienced adverse effects. Furthermore, negative symptoms were ruled out because there was no evidence of impaired communication, difficulty with abstract execution, or lack of fluency. As previous studies have shown, the psychopathological features of the present case included despair, overwhelming loss, humiliation, suicidal ideation,^14^, ^16^ decreased mental capacity, slowness of attention and thought, and fatigue (neurasthenia).^17^ However, agitation was not prominent and physical symptoms were few.^16^ In addition, there was no daily fluctuation in mood.^16^ No “psychomotor agitation,” “somatic symptoms of anxiety,” or “diurnal variation” were observed in Hamilton Depression Scale 21 items at each phase(Figure 1). Therefore, the diagnosis for this patient was PPD with relapse.

According to the WFSBP guidelines, atypical antipsychotics are appropriate as pharmacotherapy for PPD.^7^ In this case, we had the patient taper off the concomitant antidepressant and switch to monotherapy with a single antipsychotic. Thus, which antipsychotic should be administered? According to a recent review by Rooijen et al.^12^ olanzapine, aripiprazole, quetiapine, lurasidone, and amisulpride are candidates. Amisulpride could not be used by this patient because it is not marketed in Japan. Olanzapine, aripiprazole, and quetiapine were all used in the past by this patient and could not be continued due to lack of efficacy and tolerability. Pharmacologically, lurasidone has serotonin 5‐HT1A partial agonist and 5‐HT7 antagonist actions, which had antidepressant effects in functional studies in vitro.^18^ In addition, Huhn et al.^19^ reported that lurasidone is effective in acute exacerbations of depression in schizophrenia, and lurasidone significantly reduced depressive symptoms compared to placebo in a short‐term, double‐blind, randomized, controlled study of its impact on depressive symptoms.^20^ Furthermore, lurasidone is indicated for treating bipolar depression in Japan, and its anti‐hallucinogenic and delusional effects have been confirmed by using a serotonin dopamine antagonist. For these reasons, we selected lurasidone for this case. Rooijen et al.^12^ also noted that, as with typical antipsychotics, the blockade of D2 receptors is associated with worsening depression. Therefore, we maintained a low dose of lurasidone 40 mg in this patient to prevent excessive D2 blocking.

Lurasidone is a nonsedating drug and has been shown to improve Social and Occupational Functioning Assessment Scale scores.^21^ In this case, Clinical Global Impressions and Global Assessment of Functioning increased after lurasidone initiation, leading to the patient leading to employment transition assistance (Figure 1).

In summary, we presented a case of PPD with psychological symptoms of hopelessness, overwhelming loss, and suicidal ideation after 6 months of hallucinatory delusional states, in which lurasidone was effective and improved social functioning. It is crucial to diagnose PPD properly. Antipsychotics are necessary for drug treatment; however, it is controversial which drug is appropriate. Since monotherapy should be attempted as much as possible, lurasidone may be a realistic option to alleviate the depressive symptoms of PPD.

## AUTHOR CONTRIBUTIONS

YO, AN, and HK conceived and designed the contents. YO and AN wrote the paper.

## FUNDING STATEMENT

None.

## CONFLICT OF INTEREST

The authors declare no conflict of interest.

## ETHICAL APPROVAL

Not applicable.

## INFORMED CONSENT

The patient provided informed consent.

## Data Availability

This is a case report of a single patient. All the data supporting the findings are shown in the manuscript (see Figure 1).
